# Maternal placental growth factor and soluble fms-like tyrosine kinase-1 reference ranges in post-term pregnancies: A prospective observational study

**DOI:** 10.1371/journal.pone.0240473

**Published:** 2020-10-20

**Authors:** Birgitte Mitlid-Mork, Sophie Bowe, Jon M. Gran, Nils Bolstad, Jens Petter Berg, Christopher W. Redman, Anne Cathrine Staff, Meryam Sugulle

**Affiliations:** 1 Division of Obstetrics and Gynaecology, Oslo University Hospital Ullevål, Oslo, Norway; 2 Faculty of Medicine, University of Oslo, Oslo, Norway; 3 Oslo Center for Biostatistics and Epidemiology, University of Oslo and Oslo University Hospital, Oslo, Norway; 4 Division of Laboratory Medicine, Department of Medical Biochemistry, Oslo University Hospital, Oslo, Norway; 5 Nuffield Department of Obstetrics and Gynaecology, University of Oxford, Oxford, United Kingdom; Medical University Innsbruck, AUSTRIA

## Abstract

**Background:**

Post-term pregnancies have increased risks for adverse fetal and maternal outcomes. Maternal concentrations of the placenta-associated proteins placental growth factor (PlGF) and soluble fms-like tyrosine kinase-1 (sFlt-1) have been identified as predictors for preeclampsia and fetal growth restriction, both syndromes of placental dysfunction. We have proposed that low maternal circulating PlGF and increased sFlt-1 are general markers for syncytiotrophoblast stress, which increases at and beyond term, even in apparently uncomplicated pregnancies. Our aim was to establish circulating PlGF, sFlt-1, and sFlt-1/PlGF reference ranges in healthy post-term pregnancies (gestational week ≥40^+2^), comparing with healthy term pregnancies and evaluating associations between time to delivery and biomarker percentiles.

**Methods:**

Of 501 healthy, singleton post-term pregnancies prospectively recruited between September 2016 and December 2017 at our tertiary obstetric department, 426 with an uncomplicated delivery outcome contributed PlGF and sFlt-1 serum concentrations for reference range construction. A retrospective, cross-sectional, term group with an uncomplicated delivery outcome (n = 146) served as comparison. Differences in percentile values between groups and confidence intervals were calculated by quantile regression.

**Results:**

In post-term pregnancies the 5^th^, 50^th^, and 95^th^ percentiles for PlGF were: 70, 172, and 496 pg/mL; for sFlt-1: 2074, 4268, and 9141 pg/mL; and for sFlt-1/PlGF 5.3, 25.5, and 85.2. Quantile regression analyses comparing the post-term to the term group showed for PlGF a trend towards higher 10^th^ through 30^th^ percentiles, for sFlt-1 significantly higher 10^th^ through 80^th^ percentiles, and for sFlt-1/PlGF ratio significantly higher 30^th^ percentile and significantly lower 95^th^ percentile.

PlGF below the 5^th^ percentile and sFlt-1/PlGF ratio above the 95^th^ percentile was associated with shorter time to delivery (p = 0.031 and p = 0.025, respectively).

**Conclusions:**

Our findings support the concept of increasing syncytiotrophoblast stress post-term in clinically healthy pregnancies. Whether post-term dysregulated angiogenic markers reflect a biological placental clock merits further investigation.

## Introduction

Placental dysfunction is important in preeclampsia, fetal growth restriction [1], diabetic [2] and post-term pregnancies [3]. Pregnancies at term and post-term have increased risk of stillbirth [4] and neonatal morbidity [5]. Progressing placental aging may be a contributing factor to stillbirth [4,6], and is implicated in other pregnancy complications [7]. Syncytiotrophoblast stress increases at and beyond term, even in pregnancies that appear to be uncomplicated at delivery [8].

The maternal circulating placenta-associated proteins placental growth factor (PlGF) and soluble fms-like tyrosine kinase-1 (sFlt-1) are useful markers of, and risk factors for, preeclampsia [9] and fetal growth restriction (FGR) [10]. Dysregulated angiogenic factors, especially low “proangiogenic” PlGF and high “antiangiogenic” sFlt-1/PlGF ratio, predict several placental syndromes, including preeclampsia, FGR and other adverse outcomes [11–15]. Low antiangiogenic ratio (e.g sFlt-1/PlGF <38) in maternal circulation in populations with suspected preeclampsia also predict the absence of placenta-associated adverse outcomes, both preeclampsia [12] and early-onset FGR [11–15]. We have proposed that low maternal circulating PlGF may not only be a marker for preeclampsia or FGR, but also for other causes of syncytiotrophoblast stress, and thus may be utilized as a “placenta health marker” of wide clinical utility [3,8]. Maternal circulating PlGF levels in healthy pregnancies increase until around gestational week (GW) 29–30, then decrease, and show less discrimination towards term between healthy and preeclamptic pregnancies [9,16]. We have argued that this decrease in PlGF towards term is secondary to increasing cellular syncytiotrophoblast stress, which underlies the elevated stillbirth and preeclampsia rates at and after term [3].

Reference ranges for placenta-associated biomarkers beyond GW ≥40^+2^ are lacking from large populations of clinically healthy pregnancies with uncomplicated fetal and maternal outcome. In line with our hypothesis of increasing placental stress as a continuum towards the end of pregnancy, also in uncomplicated pregnancies [8], we hypothesized an increasing circulating maternal antiangiogenic biomarker pattern, with reduced PlGF, and increased sFlt-1 concentrations as well as sFlt-1/PlGF ratio in post-term pregnancies as compared to pregnancies of lower gestational age.

The primary aim of the present study was to establish gestational-age-specific reference ranges for maternal circulating concentrations of PlGF, sFlt-1, and sFlt-1/PlGF ratio in clinically healthy post-term pregnancies (GW ≥40^+2^), and to compare with those from clinically healthy term pregnancies.

## Materials and methods

### Recruitment to and clinical assessment of the prospective post-term reference group (GW ≥40^+2^)

Patients were recruited to the “Predelivery Placental Biomarkers–Pregnancy and Delivery Outcome (PREPPeD)” study from September 2016 to December 2017 at the Department of Obstetrics, Oslo University Hospital, Ullevål, delivering approximately 7100 women annually. Women with singleton pregnancies (GW ≥40^+2^), referred for routine clinical post-term evaluation, were included. Recruited women gave informed written consent. Exclusion criteria were: non Norwegian or English language, HIV and/or hepatitis, and age <18 years. According to Department protocol and national guidelines, women with low-risk pregnancies were referred to the outpatient unit between GW 41^+2^ and 41^+4^, and seen every 2–3 days until delivery or induction of labor. Induction of labor was offered to women with fetal or maternal complications, or at GW 42^+0^. Women ≥40 years of age were routinely offered an earlier appointment around GW 40^+2^ and induction of labor within GW 41^+2^. At each visit, fetal well-being was routinely assessed by cardiotocography and an ultrasound biophysical profile [17,18]. Gestational age was calculated based on routine ultrasound screening at GW 17–20 according to Norwegian national pregnancy care routine, or when not available, from last menstrual period. For in vitro fertilization, gestational age was calculated from the date of embryonal transfer. Birth weight percentile was calculated according to Norwegian population-based sex- adjusted reference ranges [19].

### Blood sampling and laboratory analyses for the prospective post-term reference group (GW ≥40^+2^)

A venous blood sample was taken at study inclusion and, if possible, daily until labor onset. The blood samples were left at room temperature for at least 30 min (max 2 hours), then centrifuged at 1800xg for 10 minutes. Serum samples were stored at -80° C until analysis (mean storage time 7.8 months). At inclusion, all women were assessed for glucose homeostasis (blood glucose and HbA1c) and kidney function (plasma creatinine).

In women with longitudinal post-term samples, the last one before labor was used to calculate the reference ranges. All samples were analyzed postpartum, blinded for clinical information at the Department of Medical Biochemistry, Oslo University Hospital, on a cobas e 801. The PIGF and sFlt-1 concentrations were quantified using the fully automated Elecsys system, according to the manufacturer´s instructions. All concentrations were within the measuring ranges of the Elecsys PlGF and sFlt-1 assays (3–10,000 pg/mL and 10–85,000 pg/mL, respectively). The coefficients of variation were ≤2.1% for PlGF and ≤1.8% for sFlt-1.

### Selection of pregnancies contributing to the construction of the reference ranges for the prospective post-term group (GW ≥40^+2^)

**Fig 1:**
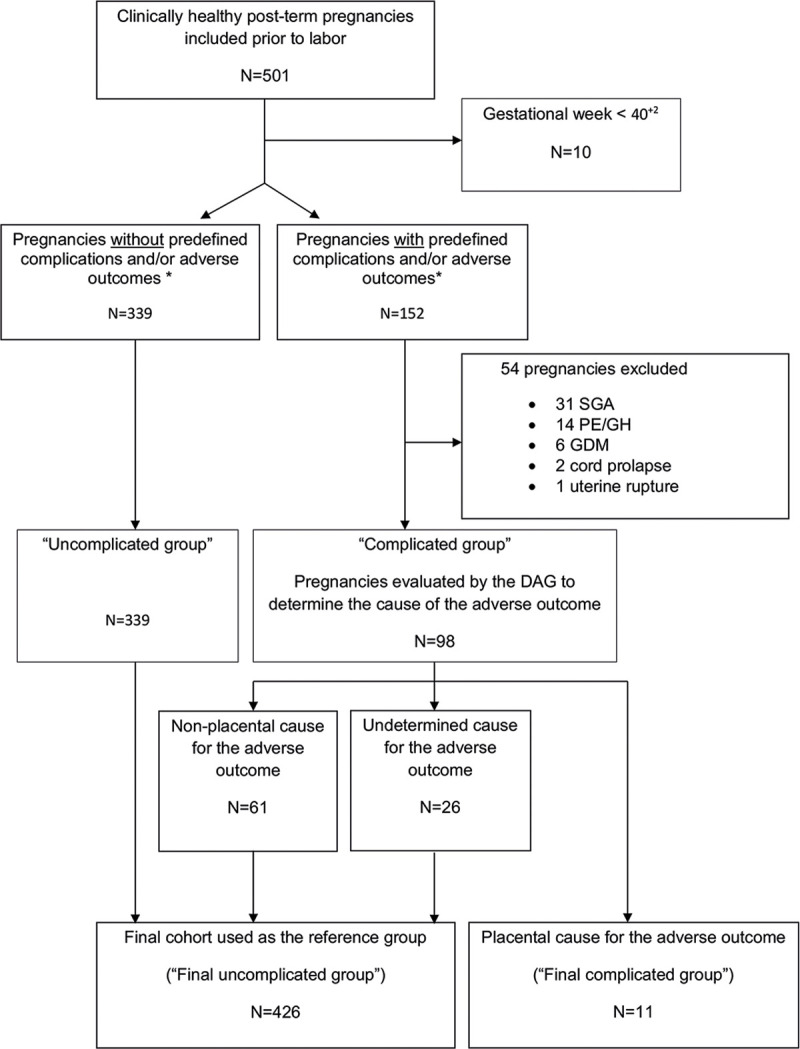
Participant flow chart of the prospective post-term group (GW 40^+2^–42^+2^), resulting in the “Final uncomplicated group” of clinically healthy pregnancies without adverse outcomes*. *PREPPeD study primary and/or secondary outcome(s). DAG, Diagnostic Advisory Group; GW, Gestational week; PE, Preeclampsia; GDM, Gestational diabetes mellitus; GH, Gestational hypertension; SGA, Small for gestational age.

Only post-term pregnancies with apparently well-functioning placentas contributed to the final maternal PlGF and sFlt-1 reference ranges. A “Diagnostic Advisory Group” (DAG), consisting of two senior consultant obstetricians, not affiliated to the PREPPeD study, and blinded for any biomarker results was established to ensure objectivity. In case of dissent regarding a pregnancy outcome categorization, a third senior consultant obstetrician reviewed and concluded the case. The DAG oversaw the process of patient grouping of all prospectively included post-term pregnancies (N = 501). Firstly, all pregnancies belonging to the "Uncomplicated group" were identified, which were the pregnancies without predefined complications and/or adverse outcomes (N = 339). Assignment to this group required thus a live born infant, no predefined pregnancy complication (birth weight <10^th^ percentile (SGA), preeclampsia (PE)/ gestational hypertension (GH)/ chronic hypertension [20], obstetric catastrophes (e.g. uterine rupture, cord prolapse or placental abruption), clinical FGR, (pre)gestational diabetes mellitus (DM) [21]), or any of the primary or secondary adverse PREPPeD delivery outcomes (as detailed in S1 Table). The pregnancies that had been recruited prior to gestational week 40^+2^ were removed from the study (N = 10; all approved by the DAG). Among the remaining 152 pregnancies with "Predefined complications and/or adverse outcomes”, 54 were excluded due to either SGA, PE, GH, gestational DM, cord prolapse, or uterine rupture (all approved by the DAG). All remaining pregnancies were assigned to the “Complicated group” (N = 98) (Fig 1). These 98 pregnancies and outcomes were scrutinized in detail by the DAG that reviewed all available clinical information including partogram details, umbilical cord blood gases and placental histology, if available. If the DAG concluded that the pregnancy outcome in the “Complicated group” was likely associated with placental dysfunction (“placental cause for the adverse outcome”; N = 11), this pregnancy was excluded from the final group contributing to the biomarker reference ranges. On the other hand, if the DAG evaluated the adverse outcome being most likely of non-placental cause (n = 61) or undetermined (n = 26), this pregnancy was included in the final group contributing to the reference values (Fig 1: “Final uncomplicated group”; N = 426).

### Comparison with data from retrospective term group (37^+0^ to 40^+0^)

Previously unpublished PlGF and sFlt-1 data from our retrospective term group (recruited to “Oslo Pregnancy Biobank” studies) served as a term comparison group to our post-term group. All women had given informed written consent. Serum from 146 apparently healthy, normotensive and euglycemic women with uncomplicated pregnancies (GW 37^+0^–40^+0^) was obtained prior to elective cesarean delivery (due to maternal request, breech presentation or repeated cesarean section) at our hospital (S2 Table). Mean sample storage time was 5.9 years before analysis for PlGF and sFlt-1, on a Roche, Elecsys 2010 Modular Analytics E170 or a cobas e601.

### Statistical analysis

Statistical analyses were performed using IBM SPSS Statistics for Windows, Version 25.0. Armonk, NY: IBM Corp. The biomarker data were skewed and thus Log transformed prior to analyses. Means were compared by two-sample T-tests and categorical variables by Chi-square tests. Differences in percentile values between two independent groups and corresponding confidence intervals (corrected for multiple testing using Bonferroni correction) were calculated by the quantreg package for quantile regression in the statistical software R [22].

### Ethics

National research ethical and institutional bodies have approved the PREPPeD (PREdelivery Placental biomarkers–Pregnancy and Delivery outcome) study (The Regional Committee for Medical and Health Research Ethics in South-Eastern Norway, ref. 2016/652), which the present study is a part of. The PREPPeD biobank is coordinated as a thematic biobank within the Oslo Pregnancy Biobank (OPB; The Regional Committee for Medical and Health Research Ethics in Eastern Norway, ref. 529–02162).

**Trial Registration**: ClinicalTrials.gov, reference number NCT03100084 (URL access on https://clinicaltrials.gov/ct2/show/NCT03100084)

## Results

In total 501 women were recruited to the prospective post-term PREPPeD group. Of these, in total 75 (15.0%) pregnancies were excluded from the “Final uncomplicated group” as follows: 10 (2.0%) were GW <40^+2^ at blood sampling, 31 (6.2%) delivered a small for gestational age (SGA) baby (<10^th^ birth weight percentile), 14 (2.8%) developed PE/GH, 6 (1.2%) developed gestational DM, 2 (0.4%) had an umbilical cord prolapse, 1 (0.2%) uterine rupture, and 11 (2.2%) had one of the primary or secondary adverse PREPPeD outcomes ascribed to probable placental dysfunction. The “Final uncomplicated group”consisted therefore of 426 clinically healthy women (Fig 1), with blood samples contributing to the post-term biomarker reference ranges in mean drawn at GW 41^+3^, and in mean 2.2 days (minimum 3 hours to maximum 11 days) before delivery.

The clinical characteristics of the “Final uncomplicated group” are listed in Table 1. Deliveries were in 68% vaginal, in 15% vaginal operative delivery, and in 17% cesarean section.

**Table 1:** Clinical characteristics of the “Final uncomplicated group” of prospectively included post-term pregnancies (GW 40^+2^–42^+2^).

Characteristics	N = 426
Nulliparous, n (%)	243 (57.0)
Maternal age in years, mean (CI)	33.5 (33.1, 33.9)
BMI 1^st^ Trimester*, mean (CI)	23.3 (23.0, 23.6)
BMI at delivery*, mean (CI)	28.7 (28.4, 29.0)
SBP at inclusion, mean (CI)	120.5 (119.6, 121.4)
DBP at inclusion, mean (CI)	76.4 (75.6, 77.1)
Blood glucose at inclusion (mmol/L), mean (CI)	4.9 (4.8, 5.0)
HbA1c at inclusion (%), mean (CI)	5.0 (4.9, 5.1)
Plasma Creatinine at inclusion (μmol/L), mean (CI)	54.6 (52.6, 56.6)
Previous CS, n (%)	31 (7.3)
Ethnicity, n (%)	
*White*	406 (95.3)
*African*	10 (2.3)
*Asian*	8 (1.9)
*Other*	2 (0.5)
Education, n (%)	
*Primary school*	2 (0.5)
*High school*	34 (8.0)
*University/college ≤4 years*	129 (30.3)
*University/college > 4 years*	261 (61.3)
Maternal smoking/snus (moist tobacco), n (%)	2 (0.5)
IVF, n (%)	19 (4.5)
GW at delivery (mean)	41^+5^
GW at blood sample closest to delivery (mean)	41^+3^
Deliveries (total), n (%)	
*Vaginal (non-operative)*	290 (68.1)
*Vacuum/forceps*	63 (14.8)
*CS*	73 (17.1)
Deliveries (Spontaneous start), n (%)	218 (51.2)
*Vaginal (non-operative)*	164 (75.2)
*Vacuum/forceps*	29 (13.3)
*CS*	25 (11.5)
Deliveries (induction of labor), n (%)	206 (48.4)
*Vaginal (non-operative)*	126 (61.2)
*Vacuum/forceps*	34 (16.5)
*CS*	46 (22.3)
Deliveries (not in labor), n (%)	2 (0.5)
*CS*	2 (100)
APGAR <4 after 1 min., n (%)	9 (2.1)
APGAR <7 after 5 min., n (%)	3 (0.7)
Placenta histology available, n (%)	91 (21.3)
Child male sex, n (%)	252 (59.2)

BMI, Body mass index; CI, 95% confidence interval; CS, Cesarean section; DBP, Diastolic blood pressure; GW, Gestational week; IVF, In vitro fertilization; SBP, Systolic blood pressure.

*Missing Data: BMI 1^st^ Trimester (2), BMI at delivery (3).

**Fig 2:**
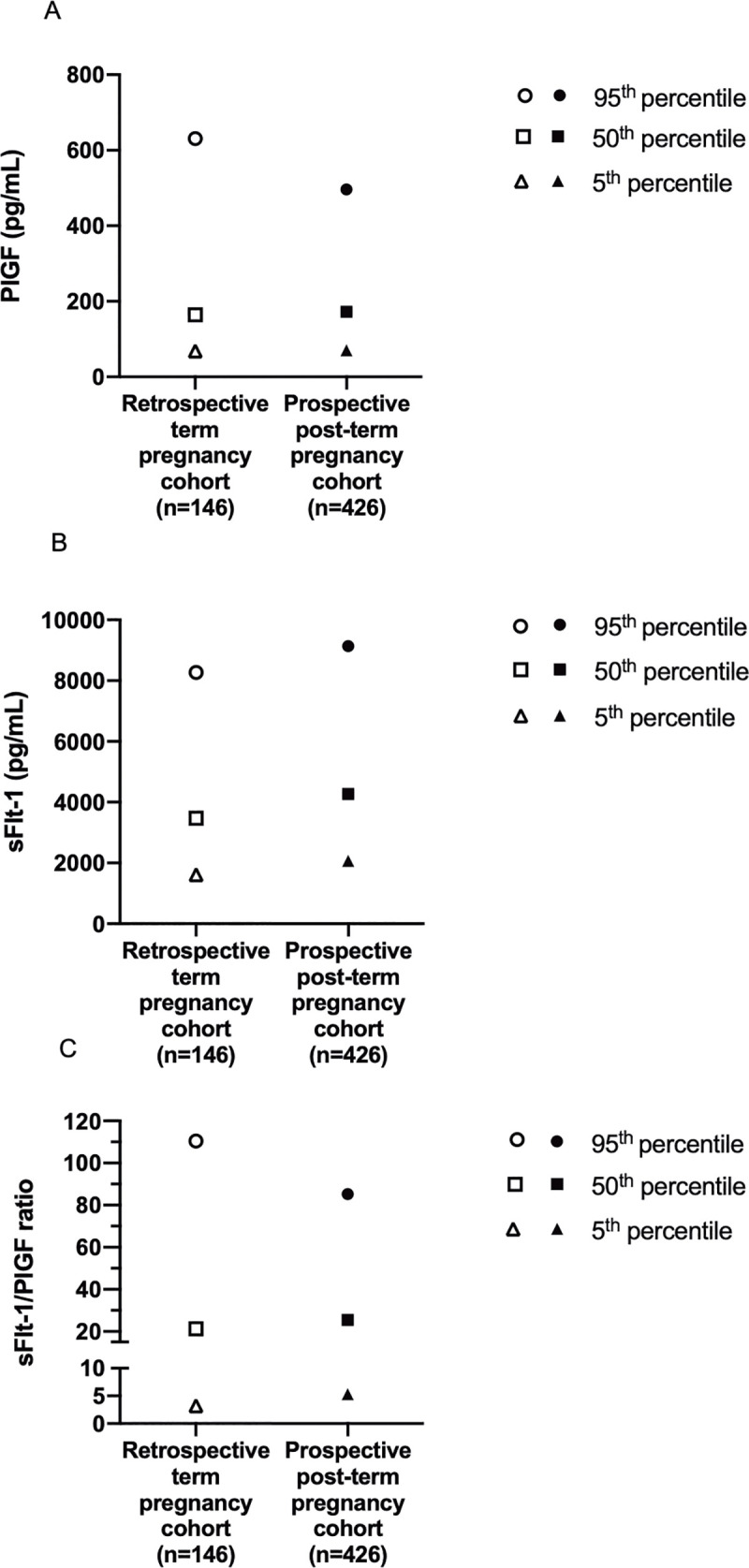
The 5^th^, 50^th^ and 95^th^ percentiles for maternal placental biomarker results for the retrospective term group as compared to the prospective post-term group. (A) Serum Placental growth factor (PlGF), (B) soluble fms-like tyrosine kinase-1 (sFlt-1), and (C) sFlt-1/PlGF ratio. Open triangles, squares, and circles represent the results of the 5^th^, 50^th^ and 95^th^ percentiles for maternal serum placental growth factor, soluble fms-like tyrosine kinase-1 and sFlt-1/PlGF ratio from the retrospective term group (GW 37^+0^–40^+0^). Filled triangles, squares, and circles represent the results of the 5^th^, 50^th^ and 95^th^ percentiles for maternal serum placental growth factor, soluble fms-like tyrosine kinase-1 and sFlt-1/PlGF ratio from our prospective post-term group (GW 40^+2^–42^+2^). Values of placental growth factor and soluble fms-like tyrosine kinase-1 are given in pg/mL.

Table 2 and Fig 2 show the reference ranges for PlGF, sFlt-1, and sFlt-1/PlGF ratio obtained from the prospective final post-term group. We found similar absolute PlGF levels for the 5^th^ and 50^th^ percentiles in the post-term group (GW 40^+2^–42^+2^) compared to our independently sampled retrospective term group (GW 37^+0^–40^+0^), but a marked reduction in the post-term group for the 95^th^ PlGF percentile (Table 2 and Fig 2A).

**Table 2:** The 5^th^, 50^th^ and 95^th^ percentiles for maternal serum placental growth factor (PlGF), soluble fms-like tyrosine kinase-1 (sFlt-1) and sFlt-1/PlGF ratio placenta-associated biomarker results in the retrospective term group and the prospective post-term group.

Placental biomarkers	Retrospective term group Gestational week 37^+0^–40^+0^ (N = 146)	Prospective post-term group Gestational week 40^+2^–42^+2^ (N = 426)
**PlGF** (pg/mL)		
5^th^ percentile	68	70
50^th^ percentile	164	172
95^th^ percentile	631	496
**sFlt-1** (pg/mL)		
5^th^ percentile	1612	2074
50^th^ percentile	3470	4268
95^th^ percentile	8267	9141
**sFlt-1/PlGF ratio**		
5^th^ percentile	3.2	5.3
50^th^ percentile	21.3	25.5
95^th^ percentile	110.5	85.2

The results from three bivariate quantile regression analyses of PlGF, sFlt-1 and sFlt-1/PlGF ratio, testing for differences in percentile values between the term and post term groups are shown in Table 3. For PlGF there was a trend towards a negative difference for the lower percentiles between the retrospective term group and the post-term group, but after correction for multiple testing, these results were no longer significant (Table 3; the 98% confidence interval (CI) includes zero).

**Table 3:** Quantile regression comparing placental biomarker percentiles between the retrospective term group (GW 37^+0^–40^+0^) and the prospective post-term group (GW 40^+2^–42^+2^).

Retrospective term group (N = 146) versus prospective post-term group (N = 426)			
Biomarker percentile	PlGF: Difference (98% CI^a^)	sFlt-1: Difference (98% CI^a^)	sFlt-1/PlGF ratio: Difference (98% CI^a^)
5^th^	-1.8 (-19.4, 6.7)	-366.0 (-1047.1, 100.9)	-2.1 (-4.4, 0.1)
10^th^	-16.1 (-25.8, 1.3)	-319.0 (-795.7, -75.7)*	-2.9 (-5.1, 0.1)
20^th^	-12.2 (-37.5, 2.7)	-484.0 (-850.7, -20.9)*	-3.1 (-6.7, 0.1)
30^th^	-14.4 (-32.9, 2.1)	-628.0 (-1093.9, -216.8)*	-4.8 (-7.7, -0.4)*
40^th^	-18.2 (-33.3, 13.1)	-830.0 (-1145.3, -383.2)*	-5.3 (-10.2, 0.2)
50^th^	-6.5 (-45.3, 34.8)	-777.0 (-1323.7, -415.2)*	-3.7 (-9.8, 1.2)
60^th^	-1.7 (-38.0, 33.1)	-1168.0 (-1584.4, -212.4)*	-4.3 (-10.4, 1.7)
70^th^	4.6 (-52.5, 88.4)	-806.0 (-1852.1, -66.7)*	-5.3 (-12.6, 11.3)
80^th^	17.7 (-48.9, 131.2)	-941.0 (-1874.1, -92.9)*	4.4 (-15.6, 23.7)
90^th^	68.9 (-52.6, 248.7)	-1000.0 (-2522.0, 577.6)	15.8 (-1.8, 49.3)
95^th^	126.5 (-65.7, 392.8)	-915.0 (-2336.6, 2061.6)	24.1 (0.1, 63.3)*

The first column indicates the biomarker percentiles being compared, the 2^nd^ through 4^th^ columns show the difference in biomarker percentiles with corresponding 98% confidence intervals for placental growth factor (PlGF), soluble fms-like tyrosine kinase-1 (sFlt-1) and sFlt-1/PlGF ratio. Positive numbers identify biomarkers percentiles higher in the term as compared to the post-term group. Negative numbers identify biomarkers percentiles higher in the post-term as compared to the term group. Significant difference between the two pregnancy groups is seen when the confidence interval does not include zero and is indicated with an asterisk.

^a^Confidence intervals (CI) are given at a 98.33% level, corresponding to 95% confidence intervals after conservative Bonferroni correction for multiple testing.

For sFlt-1, the absolute concentrations of 5^th^, 50^th^, and the 95^th^ percentiles were higher in our post-term reference group as compared to the retrospective term group (Table 2 and Fig 2B). Quantile regression analyses showed significant negative differences for sFlt-1 for the 10^th^ through 80^th^ percentiles between the retrospective term group and post-term group (Table 3).

For the sFlt-1/PlGF ratio, the absolute levels of the 5^th^ and 50^th^ percentiles were higher in the post-term group (GW 40^+2^–42^+2^) as compared to the retrospective term data (GW 37^+0^–40^+0^), but the 95^th^ percentile ratio was lower as compared to the retrospective term group (Table 2, Fig 2C). Quantile regression analyses showed a significant negative difference for sFlt-1/PlGF ratio for the 30th percentile and significant positive difference for the 95^th^ percentile between the retrospective term and the post-term group (Table 3).

The rates of post-term pregnancies with low antiangiogenic ratio (sFlt-1/PlGF <38) were similar to those in our retrospective term group (69% vs 74%, p = 0.252). Likewise, the rates of high antiangiogenic ratio (sFlt-1/PlGF >85 or sFlt-1/PlGF >110) were similar (8.9% vs 4.9%, p = 0.064 or 4.8% vs 2.1%, p = 0.082) in both groups.

When comparing the post-term pregnancies with PlGF values <5^th^ percentile with all other post-term deliveries, time to delivery was significantly lower (mean 1.4 days vs 2.2 days; p = 0.031). Similarly, post-term pregnancies with sFlt-1/PlGF ratio >95^th^ percentile had a significantly shorter time to delivery when compared to all other post-term deliveries (mean 1.4 days vs 2.2 days respectively; p = 0.025).

## Discussion

### Main findings

To the best of our knowledge, this is the first report of reference ranges for maternal circulating PlGF and sFlt-1 concentrations, as well as sFlt-1/PlGF ratio, in a prospectively collected, large group of healthy post-term pregnancies without adverse pregnancy outcomes. Two prior studies that included fewer post-term data did either not separately analyze the post-term groups [9,16], or only analyzed PlGF. Further, those studies did not exclude pregnancies with adverse pregnancy outcomes likely due to placental stress (and therefore likely altered biomarkers).

We observed a trend towards lower 70^th^ to 95^th^ percentiles of maternal circulating PlGF and significantly higher sFlt-1 10^th^ to 80^th^ percentiles in our post-term pregnancy group as compared to our retrospective term group. The antiangiogenic sFlt-1/PlGF ratio percentile was significantly higher for 30th percentile in the post-term group, but lower for the 95^th^ percentile, possibly indicating a less heterogeneous, but still elevated antiangiogenic state in clinically healthy post-term pregnancy.

### Interpretation

According to our hypothesis, cellular syncytiotrophoblast stress [3,8] and senescence [7] increase at and beyond term, with lower PlGF and higher sFlt-1 representing markers of cellular stress [3] even in pregnancies that appear to be uncomplicated. In line with this concept, we observe reduced maternal PlGF and increased sFlt-1 concentrations and sFlt-1/PlGF ratios post term in clinically healthy pregnancies without adverse fetal and maternal outcomes, when compared to term values.

The course of maternal circulating free PlGF concentrations throughout pregnancy is well described, with rising levels towards GW 29–32, and decreasing towards term [9,23], as well as a sharp increase in third trimester and towards term for sFlt-1 in a cross-sectional data set for each gestational age group [9]. Both findings were reproduced by Verlohren et al. in a longitudinal study in a demographically similar patient group to ours, applying the same analytical biomarker system [16] as us. Both Levine et al. and Verlohren et al. included fewer pregnancies ≥ GW 40^+2^ [9,16], as compared to our large post term group (n = 426), and neither of them specifically addressed post-term placenta-associated biomarker alterations in healthy pregnancies recruited in an ordinary clinical setting. The recent paper by Dunn et al reported decreasing PlGF levels by gestational age towards term and post-term [24], but did not exclude pregnancies with an adverse outcome most likely associated with placental dysfunction from their cohort contributing to their reference percentiles.

When comparing clinical characteristics, as well as sFlt-1 and PlGF concentrations from our retrospective term group (GW 37^+0^–40^+0^) with the Verlohren term group (GW > 37^+0^) [16], the data sets are comparable clinically, as were the 5^th^, 50^th^, and 95^th^ percentiles of sFlt-1, PlGF and sFlt-1/PlGF ratio. We therefore allowed us to compare our retrospective term group (GW 37^+0^–40^+0^) with the results from our prospective post-term (GW ≥40^+2^) group, assuming a reliable reflection of the longitudinal pattern changes of the angiogenic biomarkers from term to post-term in clinically healthy pregnancies. The narrower span in our post-term group between the 5^th^ and 95^th^ PlGF percentiles and observed lower PlGF levels for the 70^th^ through 95^th^ percentiles compared to the term group, although not significant after a conservative Bonferroni’s correction, are also in line with our hypothesis of an increasingly stressed placenta over the last weeks of a clinically uncomplicated pregnancy, with additional syncytiotrophoblast stress in post-term pregnancy. Our finding of even higher sFlt-1 values for all percentiles in healthy post-term pregnancies compared to term pregnancies supports our concept of increasing syncytiotrophoblast stress towards post-term pregnancy, even in those with a healthy clinical outcome.

Compared to our retrospective term pregnancy group, the 30^th^ percentile for the sFlt-1/PlGF ratio was significantly increased in the post-term group, whereas the 95^th^ percentile ratio decreased. Interestingly, we observed a trend towards higher values for the lower PlGF percentiles in the post term compared to the term group. These findings, together with the similar rate of post-term pregnancies with low antiangiogenic ratio (sFlt-1/PlGF <38) as for our retrospective term group may be due to the implicit selection bias in our study, since only the healthiest pregnancies at term (with assumedly less dysregulated angiogenic biomarkers than those already delivered) are allowed to proceed, such as the post-term women recruited to the present study. Further, there is progressive depletion of women from our study by delivery.

Our observation of a slightly shorter time to delivery in post-term healthy pregnancies with PlGF values below the 5^th^ percentile or sFlt-1/PlGF ratio above the 95^th^ is novel and not reported for healthy pregnancies before. However, further analyses in larger cohorts dichotomized into spontaneous and induced labor are warranted. Whether dysregulated angiogenic proteins at post-term might reflect increasing placental syncytiotrophoblast stress and placental membrane inflammation, promoting imminent labor onset, is an exciting concept that merits further investigation.

Our biomarker findings are consistent with increasing syncytiotrophoblast stress beyond term and imply that all pregnancies may eventually develop placental dysfunction syndromes, had the pregnancy and offspring not been “rescued” by delivery [3].

### Study strengths and limitations

Our post-term study population from a real-world unselected obstetric hospital setting was prospectively recruited and extensively clinically phenotyped, and larger than previous post-term studies [9,16,24]. Our results may therefore serve as a reference for other post-term pregnant populations. All clinical decisions were made according to Department protocol, blinded for the biomarker results. The biomarkers were analyzed collectively postpartum, blinded for clinical outcomes. All pregnancies and delivery clinical outcomes were reviewed by an obstetrical expert group blinded for biomarker results. Differences in mean storage time for the term and the post-term study groups before biomarker analyses (mean storage time 5.9 years versus 7.8 months) may be viewed as a limitation. However, PlGF and sFlt-1 serum protein levels have been proven to be stable for many years [25]. Our biobank follows strict operating procedures and samples are handled and stored in a standardized manner by a limited number of experienced study personnel. Limitations for external validity include a low ethnic heterogeneity and a large percentage of highly educated women, partly explained by the inclusion criteria (Norwegian or English language).

## Conclusions

We present reference ranges for maternal circulating PlGF and sFlt-1 in clinically healthy post-term pregnancies (GW ≥40^+2^) without placenta-related adverse delivery outcomes.

The observed lower values for the 70^th^ through 95^th^ percentile of maternal circulating PlGF as well as increased values for the 10^th^ through 80^th^ sFlt-1 percentiles in post-term compared to term pregnancies is in accordance with our hypothesis of increasing syncytiotrophoblast stress in post-term placentas.

Our novel reference ranges for placenta-associated biomarkers in healthy post-term pregnancies (GW ≥40^+2^) provide the opportunity for future testing these biomarkers as diagnostic and prognostic tools for adverse pregnancy outcomes related to placental dysfunction in post-term pregnancies. In healthy term pregnancies, low PlGF has been associated with intrapartum fetal compromise and adverse neonatal outcomes in the last weeks of pregnancy at term [26–29].

Our observation of shorter time to delivery with the highest antiangiogenic ratio and lowest PlGF percentile in the total healthy post-term pregnancies needs confirmation in further studies, but supports the biological importance of these circulating maternal biomarkers across pregnancy outcomes. We hypothesize that a”bedside” analysis of angiogenic biomarkers may in the future assist in delivery planning, such as timing of induction of labor, level of obstetric expertise needed, and delivery mode.

## Supporting information

S1 Table Primary (A: 1–9) and secondary (B: 1–2) adverse pregnancy and delivery outcomes as defined for the PREPPeD study (“Complicated group”).
(PDF)

S2 Table Clinical characteristics of the retrospective term group (GW 37^+0^–40^+0^). All patients were delivered by planned cesarean section. BMI, Body mass index; CI, 95% confidence interval; CS, Cesarean section; DBP, Diastolic blood pressure; GW, Gestational week; SBP, Systolic blood pressure. *Missing Data: BMI 1^st^ Trimester (2), BMI delivery (4), smoking (3), SBP and DBP (16), education (2), country of origin (51), parity (2), blood glucose (108).
(PDF)
