# Supplementary material for: Maternal placental growth factor and soluble fms-like tyrosine kinase-1 reference ranges in post-term pregnancies: A prospective observational study
Source: PLoS One. 2020 Oct 20;15(10):e0240473. doi: 10.1371/journal.pone.0240473 (PMC7575115; doi:10.1371/journal.pone.0240473)
Supplement: S1 Table — Primary (A: 1–9) and secondary (B: 1–2) adverse pregnancy and delivery outcomes as defined for the PREPPeD study (“Complicated group”). (PDF) [file pone.0240473.s001.pdf]

S1 Table. Primary (A: 1-9) and secondary (B: 1-2) adverse pregnancy and delivery outcomes as defined for the PREPPeD study ("Complicated group")

**A: Primary adverse outcomes** (Either of the composite adverse outcomes 1-9):	
1	Fetal acidemia, evaluated by:<br>A. Umbilical cord blood gases (pH, base deficit (BD) as used by our Oxford collaborators):<br>I. In neonates delivered by cesarean section (CS) without labor (defines as absence of regular uterine contractions): umbilical artery blood (transporting blood from the fetus to the placenta) pH <7.13 and arterial BD >10.0<br>II. In neonates from labored delivery (regardless of subsequent method, vaginal or CS): Umbilical artery blood pH <7.05 and arterial BD>14<br>OR<br>B. Umbilical artery blood lactate (or venous if arterial blood not available) above reference level for respective gestational age[1]
2	Newborn low Apgar<br>A. <4 at 1 minute<br>OR<br>B. <7 at 5 minutes (any newborn intubated at this time point will be registered as low Apgar at 5 minutes, as Apgar cannot be assessed in assisted ventilation
3	Newborn asphyxia: defines as fetal acidemia (#1 above) AND newborn low Apgar (#2 above)
4	Rate of intrauterine fetal demise/intra-/postpartum fetal death
5	Neonatal intubation/mechanical ventilation >6 hours
6	Meconium aspiration syndrome
7	Neonatal hypoxic-ischemic encephalopathy
8	Therapeutic hypothermia of the neonate
9	Rate of acute cesarean section (due to suspected fetal distress)
**B: Secondary adverse outcomes**	
1	Rates of operative vaginal deliveries (forceps/vacuum/combined; due to suspected fetal distress
2	Pathological placenta histology findings

1. Wiberg N, Källén K, Herbst A, Åberg A, Olofsson P. Lactate concentration in umbilical cord blood is gestational age-dependent: a population-based study of 17 867 newborns. *BJOG*. 2008;115:704-9.
