# Supplementary material for: Maternal placental growth factor and soluble fms-like tyrosine kinase-1 reference ranges in post-term pregnancies: A prospective observational study
Source: PLoS One. 2020 Oct 20;15(10):e0240473. doi: 10.1371/journal.pone.0240473 (PMC7575115; doi:10.1371/journal.pone.0240473)
Supplement: S2 Table — All patients were delivered by planned cesarean section. BMI, Body mass index; CI, 95% confidence interval; CS, Cesarean section; DBP, Diastolic blood pressure; GW, Gestational week; SBP, Systolic blood pressure. *Missing Data: BMI 1st Trimester (2), BMI delivery (4), smoking (3), SBP and DBP (16), education (2), country of origin (51), parity (2), blood glucose (108). (PDF) [file pone.0240473.s002.pdf]

S2 Table. Clinical characteristics of the retrospective term group (GW 37^{+0}-40^{+0})

Characteristics	N=146
Nulliparous*, n (%)	59 (40.4)
Maternal age in years, mean (CI)	33.3 (32.7, 34.0)
BMI 1st Trimester*, mean (CI)	23.5 (22.9, 24.2)
BMI at delivery*, mean (CI)	28.7 (28.1, 29.3)
SBP at inclusion*, mean (CI)	126 (123, 128)
DBP at inclusion*, mean (CI)	75 (73, 77)
Blood glucose closest to delivery (mmol/L)*, mean (CI)	4.0 (3.8, 4.1)
Ethnicity*, n (%)	
Caucasian	81 (55.5)
African	3 (2.1)
Asian	2 (1.4)
Other	9 (6.2)
Education*, n (%)	
Primary school	0 (0)
High school	9 (6.2)
University/college ≤4 years	13 (8.9)
University/college > 4 years	27 (18.5)
Unknown	95 (65.1)
Maternal smoking/snus, n (%)	4 (2.7)
GW at delivery (mean)	38^{+6}
GW at blood sample closest to delivery (mean)	38^{+6}
APGAR <4 after 1 min., n (%)	0 (0)
APGAR <7 after 5 min., n (%)	0 (0)

Child male sex, n (%)	78 (53.4)

All patients were delivered by planned cesarean section.

BMI, Body mass index; CI, 95% confidence interval; CS, Cesarean section; DBP, Diastolic blood pressure; GW, Gestational week; SBP, Systolic blood pressure

*Missing Data: BMI 1st Trimester (2), BMI delivery (4), smoking (3), SBP and DBP (16), education (2), country of origin (51), parity (2), blood glucose (108)
